# The curvilinear associations between Life’s Crucial 9 and frailty: cross-sectional study of NHANES 2003 - 2023

**DOI:** 10.3389/fragi.2025.1528338

**Published:** 2025-06-06

**Authors:** Bo Wang, Chunqi Jiang, Ning Wang, Yinuo Qu, Jun Wang, Guang Zhao, Xin Zhang

**Affiliations:** ^1^ Pediatrics Department, Central Hospital of Jinan City, Jinan, Shandong, China; ^2^ Affiliated Hospital of Shandong University of Traditional Chinese Medicine, Jinan, Shandong, China; ^3^ Basic Medical College, Shandong University of Traditional Chinese Medicine, Jinan, Shandong, China

**Keywords:** life’s crucial 9, frailty, curve, NHANES, cross-sectional study

## Abstract

**Background:**

Frailty not only affects disease survival rates but also the quality of life. The Life’s Crucial 9 (LC9) is a recently proposed cardiovascular health risk score that incorporates mental health along with Life’s Essential 8 (LE8). The association between LC9 and frailty has not yet been reported. This study aims to explore the link between LC9 scores and levels of frailty.

**Methods:**

We used a weighted multiple logistic regression model to evaluate the relationship between Life’s Essential 8 (LE8) and LC9 with frailty, and conducted trend tests to assess the stability of this association. Additionally, we employed smooth curve fitting to explore the potential curvilinear relationship between LE8 and LC9 with frailty. To identify inflection points, we applied recursive partitioning algorithms in conjunction with a two-stage linear regression model. Stratified analyses were performed to examine heterogeneity within various populations.

**Results:**

Our study included a total of 28,557 participants. In the regression model that accounted for all covariates, the odds ratios (ORs) for the association of LE8 and LC9 scores with frailty were 0.95 (95% CI: 0.94, 0.95) and 0.93 (95% CI: 0.93, 0.93), respectively, indicating a significantly stronger negative correlation with LC9 scores. Sensitivity analysis confirmed the robustness of this relationship. Smooth curve fitting revealed a nonlinear correlation between LE8 and LC9 scores and the degree of frailty. Further analysis using a two-piecewise linear regression model identified inflection points at 53.12 for LE8 and 68.89 for LC9. Below these thresholds, both LE8 and LC9 demonstrated a significant negative association with frailty. However, above these points, the strength of the negative correlation was somewhat reduced but remained statistically significant. In stratified analyses, both LE8 and LC9 exhibited significant negative associations with frailty, with LC9 showing a more pronounced relationship. Significant interaction effect was detected within the education level groups.

**Conclusion:**

We found a curvilinear relationship between LE8, LC9 and frailty, and the relationship between LC9 and frailty was more significant. This implies that LC9 can facilitate the early and precise identification of individuals at high risk of frailty, thereby providing a foundation for the development of targeted intervention strategies.

## 1 Introduction

Frailty emerges as a critical health issue on a global scale, defined by heightened susceptibility to stress, a diminished capacity in multiple physiological systems, and a degradation in functional capabilities (Hoogendijk et al., 2019). It serves as a biomarker of aging, with effects that span beyond physical health to include cognitive, emotional, and social aspect (Pek et al., 2020). This multifaceted syndrome is tied to the impairment of physiological systems, its incidence escalating with advancing age and adversely affecting survival across all age brackets (Song et al., 2010). Approximately 26.8% of the senior population grapples with frailty, which correlates with an elevated risk of negative health consequences such as disabilities, hospital admissions, mortality, progression of cardiovascular diseases, and the incidence of major cardiovascular events (Veronese et al., 2021; Qin and Zheng, 2023; Damluji et al., 2021). In response to these ramifications, the International Symposium on Frailty and Sarcopenia Research stresses the imperative of prompt detection, comprehensive assessment, and strategic management of frailty to augment life expectancy and enhance the quality of life (Dent et al., 2019).

Life’s Crucial 9 (LC9) represents an advancement in the American Heart Association’s (AHA) suite of health assessment tools, expanding on the earlier Life’s Simple 7 (LS7) and Life’s Essential 8 (LE8) metrics (Ge et al., 2024). LS7, launched in 2010, concentrated on seven pivotal health behaviors and factors, such as smoking, diet, physical activity, body mass index, blood pressure, cholesterol, and fasting glucose levels (Lloyd-Jones et al., 2010). LE8, a subsequent revision of LS7, enhanced the assessment by adding sleep as a critical health metric and refining the scoring algorithm for the initial seven components (Lloyd-Jones et al., 2022). LC9 elevates this framework further by incorporating psychological health, with a specific focus on depression, into the LE8 construct. This innovative metric acknowledges the profound influence of mental health, particularly depression, on cardiovascular health—a dimension that was not comprehensively considered in prior models (Gaffey et al., 2024).

Currently, research on the LC9 score is still in its nascent stages. However, prior studies have shown that higher LE8 scores are associated with a reduced likelihood of frailty symptoms in cancer patients (Qiu et al., 2024). Additionally, Suo X et al. (2025) have demonstrated that frailty is linked to a higher risk of depression. Similarly, a cross-sectional study by Yang L et al. (2025) revealed that depressive symptoms can increase the risk of frailty in older adults. Despite these findings, the relationship between LC9 scores and frailty symptoms remains unexplored. This study aims to examine the correlation between LC9 scores and frailty, offering novel insights and strategies for continuous health management and lifestyle interventions for individuals with frailty. interventions for individuals with frailty.

## 2 Materials and methods

### 2.1 Study participants

**FIGURE 1:**
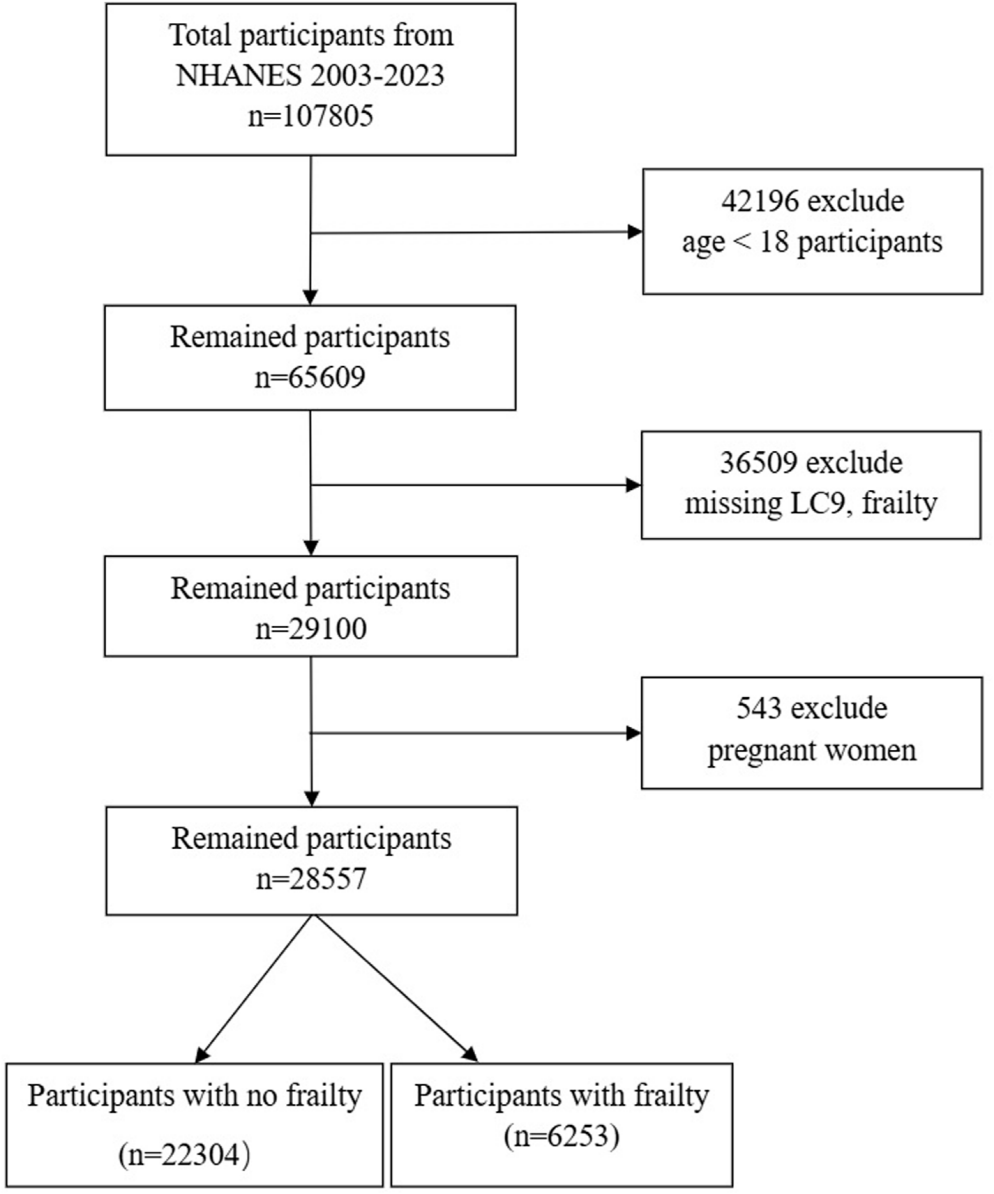
Flow chart of sample selection from the 2003–2023. Abbreviations: LC9, Life’s Crucial 9.

Our investigation drew on data from the National Health and Nutrition Examination Survey (NHANES) in the United States, covering a period from 2003 to 2023 and including ten survey cycles. The NHANES compiles a wide array of data, including demographic details, lifestyle elements, self-reported health measures, and blood biochemistry assessments. The data gathering process involves in-home interviews, visits to mobile examination centers, and lab tests. This dataset is accessible to researchers without the necessity for specific permissions. The research protocol was endorsed by the National Center for Health Statistics’ Institutional Review Board, with all participants offering their written consent. Personal identifiers were anonymized to protect privacy. For the data preparation stage, we excluded those under 18, summing up to 28,047 individuals. Another 13,043 individuals were left out due to the absence of frailty and LC9 data. Pregnant women, numbering 543, were also excluded. Ultimately, our study involved 28,557 participants, as illustrated in Figure 1.

### 2.2 Study variables

#### 2.2.1 Definition of frailty

Frailty is diagnosed using the frailty index proposed by Searle and his colleagues, which is a quantitative measure that encapsulates the accumulation of deficits across multiple systems (Searle et al., 2008). This index includes 49 variables from various domains, including cognition, dependency, depressive symptoms, comorbidities, general health status and hospital utilization, physical function, and anthropometric measures, as well as laboratory test values (Hakeem et al., 2021). These variables, which indicate health deficits, typically increase with age and do not reach premature saturation. All deficits, whether ordinal, continuous, or binary, are represented as values between 0 (absence) and 1 (maximum presence) based on their severity. The frailty index is calculated by dividing the actual scores of the deficits by 49 (the total number of included deficits). A frailty index score of 0.21 has been established as the threshold for identifying individuals with frailty, with higher scores indicating a greater degree of frailty (Blodgett et al., 2015).

#### 2.2.2 Definition of LC9

The LC9 score is calculated as the unweighted average of the nine component indicators, which include the LE8 score and the PHQ-9 score. Detailed instructions for calculating the LC9 score for each participant using the NHANES database are provided in Supplementary Material. The LC9 score is calculated as the average of two components: the LE8 score and the score from the PHQ-9 (Ge et al., 2024). The LE8 score is based on metrics introduced by AHA in 2022 (Lloyd-Jones et al., 2022). It includes four health behaviors—diet, physical activity, nicotine exposure, and sleep duration—along with four health factors: body mass index (BMI), non-HDL cholesterol, blood glucose, and blood pressure. Each of these eight Cardiovascular Health metrics is scaled from 0 to 100, with the overall LE8 score representing the mean of these individual metrics. The PHQ-9 score categorizes depression levels into five ranges: 100, 75, 50, 25, and 0, which correspond to the PHQ-9 score ranges of 0–4, 5–9, 10–14, 15–19, and 20–27, respectively (Zhang et al., 2023). Dietary assessments are based on quintiles of the Healthy Eating Index (HEI-2015) (Krebs-Smith et al., 2018), which utilizes dietary information obtained from two 24-h dietary recalls. This data is combined with the United States Department of Agriculture’s Food Pattern Equivalents to compute the HEI-2015 score. Physical activity levels, nicotine exposure, sleep duration, and diabetes history are determined through standardized self-report questionnaires. During physical examinations, height and weight are measured, and blood pressure is recorded. BMI is calculated by dividing weight in kilograms by the square of height in meters. Blood samples are collected for centralized laboratory analysis to evaluate lipid profiles, fasting blood glucose, and glycated hemoglobin levels.

#### 2.2.3 Assessment of other variables

The covariates considered in our study were collected by the CDC using computer-assisted personal interviews and mobile examination centers. These include age, gender, race, education level, marital status, poverty-income ratio, smoking status, alcohol consumption, recreational activity, diabetes, hypertension, cardiovascular disease, and high-density lipoprotein cholesterol. Race was categorized as Mexican American, non-Hispanic white, non-Hispanic black, other Hispanic, or other race. Education levels were divided into less than high school, high school, and more than high school; marital status into never married, married/living with partner, divorced/widowed/separated; smoking status was divided into three distinct groups: ‘Never’ defined as less than 100 cigarettes in one’s lifetime; ‘Former’ for those with a history of smoking but have quit; and ‘Now’ designated for those who continue to smoke (Chambers et al., 2011). Participation in recreational activities was binary, recorded as ‘Yes’ or ‘No’. Diabetes diagnosis, including pre-diabetes, was based on meeting at least one of the following criteria: 1. Fasting blood glucose above 7.0 mmol/L; 2. Hemoglobin A1c (HbA1c) 6.5% or higher; 3. Random blood glucose level of at least 11.1 mmol/L; 4. Blood glucose level of 11.1 mmol/L or higher after a 2-h oral glucose tolerance test (OGTT); 5. A formal diagnosis of diabetes by a healthcare provider; 6. Impaired fasting glucose ranging from 6.11 to 7.0 mmol/L or impaired glucose tolerance, OGTT levels between 7.7 and 11.1 mmol/L. Hypertension was determined by one or more of the following conditions: 1. Systolic blood pressure reading of 140 mmHg or higher; 2. Diastolic blood pressure of 90 mmHg or higher; 3. Current use of antihypertensive medication; 4. Self-reported hypertension. Alcohol consumption levels were classified as follows: ‘heavy’ drinking characterized by women consuming three or more drinks per day or four or more drinks on a single occasion; men consuming four or more drinks per day or five or more drinks on a single occasion, with at least five heavy drinking days per month. ‘Moderate’ drinking was defined as women consuming two drinks per day and men three drinks per day, with at least two heavy drinking days per month. ‘Mild’ drinking referred to women consuming one drink per day and men two drinks per day. ‘Never’ drinking referred to those who had less than 12 drinks in their lifetime, while ‘Former’ drinkers were those with a history of drinking but no longer consumed alcohol. Cardiovascular disease (CVD) was determined through a medical history questionnaire, recording whether participants had been diagnosed with coronary artery disease, congestive heart failure, or had a history of heart attack (Xu et al., 2020).

### 2.3 Statistical analysis

Through the appropriate weighting of study data, the representativeness of our sample to the population was enhanced. We managed missing data by imputation, utilizing predictive mean matching for continuous variables and logistic regression for binary variables. Participants were divided into two groups—those with frailty and those without—based on their initial characteristics. Continuous variables are expressed as mean values with standard error, while categorical variables are presented as proportions of the entire sample. To explore the link between LE8 and LC9 scores and frailty, we utilized weighted logistic regression analysis, with outcomes reported as odds ratios (ORs) along with their corresponding 95% confidence intervals (95% CI). A linear trend analysis was conducted to verify the consistency of the associations observed between LE8, LC9, and frailty. Curve fitting was then applied to identify any non-linear trends. Two-piecewise linear regression models were established using recursive algorithms to pinpoint inflection points. Stratified analyses were conducted to reveal variations in the relationships between LE8, LC9, and frailty among different demographic groups. Statistical analyses were carried out using R (version 3.5.3) and EmpowerStats software (http://www.empowerstats.com), with statistical significance set at a P-value of less than 0.05.

## 3 Results

### 3.1 Baseline characteristics

Table 1 delineates the baseline characteristics of participants, contrasting those without frailty (n = 22,304) with those who are frail (n = 6,253). The average age of participants identified as frail is significantly higher at 57.50 years, surpassing the 46.34 years observed in the non-frail cohort. There is a notable predominance of females within the frail group, constituting 61.83%, in contrast to the 49.65% found in the non-frail group. Participants with frailty are more likely to have lower levels of education, to be divorced, widowed, or separated, and to report no engagement in recreational activities. Moreover, the incidence of chronic conditions such as diabetes, hypertension, cardiovascular disease, and depression is markedly higher among the frail population. Relative to their non-frail counterparts, individuals with frailty exhibit a lower income to poverty ratio, a higher BMI, elevated TG, and reduced sleep duration.

**TABLE 1:** Baseline characteristics of participants.

Characteristic	Without frailty (n = 22,304)	With frailty (n = 6,253)	P Value
Age (year)	46.34 ± 16.37	57.50 ± 15.18	<0.0001
Sex (%)			<0.0001
Female	49.65	61.83	
Male	50.35	38.17	
Race/ethnicity (%)			<0.0001
Mexican American	7.99	6.07	
Non-Hispanic White	70.28	67.58	
Non-Hispanic Black	9.87	15.24	
Other Hispanic	5.08	5.13	
Other Race	6.78	5.99	
Marry status (%)			<0.0001
Never married	18.22	10.82	
Married/Living with partner	66.38	56.79	
Divorced/Widowed/Separated	15.40	32.38	
Education status (%)			<0.0001
Less than high school	3.75	7.90	
High school	31.10	43.09	
More than high school	65.15	49.00	
Recreational activity (%)			<0.0001
No	40.23	69.05	
Yes	59.77	30.95	
Drinking status (%)			<0.0001
Never	10.37	11.92	
Mild	38.73	32.62	
Moderate	18.50	13.78	
Heavy	21.12	16.22	
Former	11.28	25.47	
Smoking status (%)			<0.0001
Never	57.84	41.17	
Now	17.68	26.69	
Former	24.48	32.14	
Diabetes (%)			<0.0001
No	82.59	52.97	
Yes	17.41	47.03	
Hypertension (%)			<0.0001
No	67.95	28.86	
Yes	32.05	71.14	
CVD (%)			<0.0001
No	95.77	68.74	
Yes	4.23	31.26	
Depression (%)			<0.0001
No	97.35	68.22	
Yes	2.65	31.78	
Income to poverty ratio	3.22 ± 1.60	2.39 ± 1.58	<0.0001
BMI (kg/m2)	28.58 ± 6.41	32.08 ± 8.27	<0.0001
TC (mmol/L)	5.04 ± 1.03	4.96 ± 1.24	<0.0001
TG (mmol/L)	1.36 ± 1.05	1.72 ± 1.71	<0.0001
LC9	72.24 ± 12.66	57.09 ± 13.01	<0.0001
LE8	70.13 ± 13.63	56.26 ± 13.70	<0.0001
Sleep duration (h)	7.13 ± 1.30	6.98 ± 1.84	<0.0001
HbA1c (%)	5.51 ± 0.77	6.13 ± 1.30	<0.0001

Average values ±standard error are provided for continuous variables, with p values derived from the weighted linear regression model. Percentages are used to represent categorical variables, and their p values are obtained through a weighted chi-square test. Abbreviations: BMI: body mass index; CVD: cardiovascular disease; TG, triglycerides; TC, total cholesterol; HbA1c, Glycosylated hemoglobin; LE8, Life’s Essential 8; LC9, Life’s Crucial 9.

### 3.2 Association between LC9 and frailty

Table 2 elucidates the relationship between LE8 and LC9 scores and the risk of frailty within three progressively adjusted models. Model 1 serves as the baseline without any adjustments, while Model 2 incorporates adjustments for sex, age, and race. Model 3 further refines these adjustments by including additional factors such as the family income to poverty ratio, education level, marital status, and drinking behavior. In the fully adjusted Model 3, the odds ratio (OR) for the association between LE8 and frailty is 0.95 (95% CI: 0.94, 0.95), and for LC9, it is 0.93 (95% CI: 0.93, 0.93). The congruence of findings across the models underscores the robust link between LE8 and LC9 scores and the risk of frailty, with LC9 exhibiting a marginally stronger relationship. For both LE8 and LC9, higher quartiles are correlated with a decreased risk of frailty, as evidenced by a significant downward trend in ORs from Q1 to Q4.

**TABLE 2:** The association between LE8, LC9 and frailty.

Exposure	Model 1 OR (95% CI)	P Value	Model 2 OR (95% CI)	P Value	Model 3 OR (95% CI)	P Value
LE8	0.93 (0.93, 0.94)	<0.0001	0.94 (0.94, 0.94)	<0.0001	0.95 (0.94, 0.95)	<0.0001
LE8 quartile						
Q1	Reference		Reference		Reference	
Q2	0.41 (0.38, 0.44)	<0.0001	0.43 (0.40, 0.47)	<0.0001	0.48 (0.44, 0.52)	<0.0001
Q3	0.19 (0.17, 0.20)	<0.0001	0.22 (0.20, 0.24)	<0.0001	0.25 (0.23, 0.27)	<0.0001
Q4	0.07 (0.07, 0.08)	<0.0001	0.10 (0.09, 0.11)	<0.0001	0.12 (0.11, 0.14)	<0.0001
P for trend	<0.0001		<0.0001		<0.0001	
Sex						
Female	0.93 (0.93, 0.93)	<0.0001	0.94 (0.93, 0.94)	<0.0001	0.94 (0.94, 0.95)	<0.0001
Male	0.94 (0.94, 0.94)	<0.0001	0.94 (0.94, 0.95)	<0.0001	0.95 (0.94, 0.95)	<0.0001
Age						
60<	0.93 (0.93, 0.93)	<0.0001	0.93 (0.93, 0.93)	<0.0001	0.94 (0.93, 0.94)	<0.0001
= >60	0.95 (0.94, 0.95)	<0.0001	0.94 (0.94, 0.95)	<0.0001	0.95 (0.95, 0.95)	<0.0001
BMI						
<30	0.93 (0.93, 0.94)	<0.0001	0.94 (0.94, 0.94)	<0.0001	0.95 (0.95, 0.95)	<0.0001
= >30	0.94 (0.93, 0.94)	<0.0001	0.94 (0.94, 0.94)	<0.0001	0.95 (0.94, 0.95)	<0.0001
LC9	0.92 (0.92, 0.92)	<0.0001	0.92 (0.92, 0.93)	<0.0001	0.93 (0.93, 0.93)	<0.0001
LC9 quartile						
Q1	Reference		Reference		Reference	
Q2	0.34 (0.31, 0.36)	<0.0001	0.35 (0.32, 0.38)	<0.0001	0.37 (0.34, 0.41)	<0.0001
Q3	0.15 (0.14, 0.16)	<0.0001	0.17 (0.15, 0.18)	<0.0001	0.19 (0.18, 0.21)	<0.0001
Q4	0.05 (0.04, 0.06)	<0.0001	0.06 (0.06, 0.07)	<0.0001	0.08 (0.07, 0.09)	<0.0001
P for trend	<0.0001		<0.0001		<0.0001	
Sex						
Female	0.92 (0.91, 0.92)	<0.0001	0.92 (0.92, 0.92)	<0.0001	0.93 (0.92, 0.93)	<0.0001
Male	0.93 (0.92, 0.93)	<0.0001	0.93 (0.92, 0.93)	<0.0001	0.93 (0.93, 0.94)	<0.0001
Age						
60<	0.92 (0.91, 0.92)	<0.0001	0.91 (0.91, 0.92)	<0.0001	0.92 (0.92, 0.92)	<0.0001
= >60	0.93 (0.93, 0.94)	<0.0001	0.93 (0.93, 0.94)	<0.0001	0.94 (0.93, 0.94)	<0.0001
BMI						
<30	0.92 (0.92, 0.92)	<0.0001	0.93 (0.92, 0.93)	<0.0001	0.93 (0.93, 0.94)	<0.0001
= >30	0.92 (0.92, 0.92)	<0.0001	0.92 (0.92, 0.93)	<0.0001	0.93 (0.93, 0.94)	<0.0001

Model 1: no adjustment.

Model 2: adjusted for sex, age and race.

Model 3: adjusted for sex, age, race, family income to poverty ratio, education level, marriage status, and drinking status.

In subgroup analyses stratified by age, BMI, or gender, the relationship between LE8, LC9 and frailty was assessed without adjusting for the stratification variables. Abbreviations: BMI, body mass index; LE8, Life’s Essential 8; LC9, Life’s Crucial 9; OR, odds ratios; CI, confidence intervals.

Subgroup analyses stratified by sex, age, and BMI demonstrate that the association between LE8 and LC9 scores and frailty remains consistent among different demographic subgroups. In the gender-stratified analysis, the LE8 score demonstrated a more pronounced association with frailty among females, with an OR of 0.94 (95% CI: 0.94, 0.95), compared to males, where the OR was 0.95 (95% CI: 0.94, 0.95). This suggests a slightly stronger relationship for females in the context of LE8 score and frailty. In contrast, for the LC9 score, no significant gender differences were noted in its association with frailty. When examining the data by age, both LE8 and LC9 scores revealed a more robust association with frailty in individuals under the age of 60. As for BMI stratification, there was no discernible difference in the association between LE8 and LC9 scores and frailty.

**FIGURE 2:**
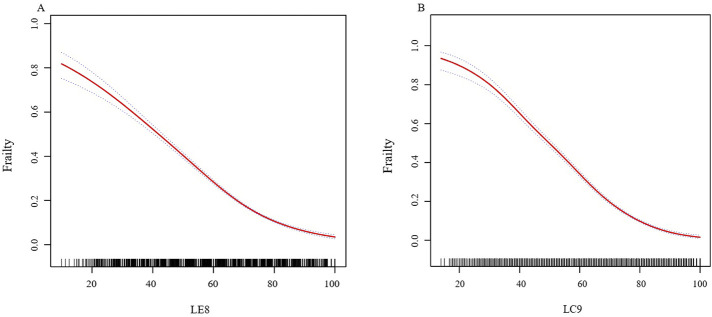
**(A)** The nonlinear associations between LE8 and frailty. **(B)** The nonlinear associations between LC9 and frailty. We adjusted for sex, age, race, family income to poverty ratio, education level, marriage status, and drinking status. Abbreviations: LE8: Life’s Essential 8; LC9: Life’s Crucial 9.

In further analysis, smooth curve fitting confirmed the curvilinear relationship between LE8, LC9, and CKD, as illustrated in Figure 2. Table 3 utilizes a two-piecewise linear regression model to investigate the threshold effects of LE8 and LC9 scores on frailty. The two-piecewise linear model identifies inflection points at 53.12 for LE8 and 68.89 for LC9. Below these thresholds, the ORs for LE8 and LC9 are 0.96 (95% CI: 0.95, 0.96) and 0.93 (95% CI: 0.93, 0.94), respectively, suggesting a continued negative association with frailty. However, above the inflection points, the ORs for LE8 and LC9 decrease to 0.94 (95% CI: 0.94, 0.95) and 0.92 (95% CI: 0.91, 0.93), respectively, indicating a slightly diminished protective effect against frailty. The association between LC9 and frailty is more pronounced than that between LE8 and frailty, both before and after the inflection points.

**TABLE 3:** The threshold effects of LE8, LC9 on frailty were analyzed using a two-piecewise linear regression model.

Outcome	LE8 OR (95% CI)	P Value	LC9 OR (95% CI)	P Value
Fitting by standard linear model	0.95 (0.94, 0.95)	<0.0001	0.93 (0.93, 0.93)	<0.0001
Fitting by two-piecewise linear model				
Inflection point (K)	53.12		68.89	
<K	0.96 (0.95, 0.96)	<0.0001	0.93 (0.93, 0.94)	<0.0001
>K	0.94 (0.94, 0.95)	<0.0001	0.92 (0.91, 0.93)	<0.0001
Log-likelihood ratio	<0.001		0.001	

We adjusted for sex, age, race, family income to poverty ratio, education level, marriage status, and drinking status. Abbreviations: LE8, Life’s Essential 8; LC9, Life’s Crucial 9; OR, odds ratios; CI, confidence intervals.

Figure 2 The nonlinear associations between LE8, LC9, and frailty. We adjusted for sex, age, race, family income to poverty ratio, education level, marriage status, and drinking status.

Table 4 provides the results of the subgroup analysis, which investigates the potential interaction effects of LC9 on frailty across different demographic groups. The findings reveal that the association between LC9 and frailty is more pronounced among participants with less than a high school education compared to those with a high school education or higher. This suggests a significant interaction effect within the education level groups.

**TABLE 4:** Subgroup analysis.

Subgroup	OR, (95% CI)	P Value	P Interaction
Drinking status			0.3240
Never	0.92 (0.92, 0.93)	<0.0001	
Mild	0.93 (0.93, 0.94)	<0.0001	
Moderate	0.93 (0.93, 0.93)	<0.0001	
Heavy	0.93 (0.92, 0.94)	<0.0001	
Former	0.93 (0.92, 0.94)	<0.0001	
Education status			0.0083
Less than high school	0.92 (0.92, 0.93)	<0.0001	
High school	0.94 (0.93, 0.94)	<0.0001	
More than high school	0.93 (0.92, 0.93)	<0.0001	
Marriage status			0.4877
Never married	0.93 (0.93, 0.94)	<0.0001	
Married/Living with partner	0.93 (0.93, 0.93)	<0.0001	
Divorced/Widowed/Separated	0.93 (0.92, 0.93)	<0.0001	

We adjusted for factors such as gender, age, race, family income-to-poverty ratio, educational level, marital status, and alcohol consumption, but we did not adjust for the subgroups themselves. Abbreviations: OR: odds ratios; CI: confidence intervals.

## 4 Discussion

Using data from 10 cycles of the NHANES spanning from 2003 to 2023, our research establishes that higher LC9 scores correlate with a reduced likelihood of frailty. We have conducted an in-depth examination of the relationship between both LE8 scores and LC9 scores with frailty. Our findings reveal a negatively correlated, curved association between these scores and frailty, with a notable inflection point where the relationship attenuates but remains statistically significant. Moreover, the LC9 score exhibits a higher inflection point, and its association with frailty is more substantial than that of the LE8 score both before and after the inflection point.

The LC9 score, which encompasses psychological health in addition to the components of LE8, represents an innovative approach in health metrics. Our research stands at the forefront as the initial investigation into the relationship between LC9 and frailty. The research on the association between LE8 and frailty is still in its infancy. Despite the limited number of studies, a consistent conclusion has emerged: higher LE8 scores are significantly linked to a reduced risk of frailty, with a clear dose–response relationship. Ma Q et al. demonstrated in a study of middle-aged and older adults in the United States that an increase in LE8 scores significantly reduces the risk of frailty, with a distinct negative dose–response trend. Qiu X et al. further confirmed that this conclusion holds true among cancer survivors. However, existing studies generally lack precise identification of the inflection points in the dose–response curve and have not systematically explored the dynamic changes in the relationship between variables before and after these inflection points. Additionally, most studies have focused on specific populations, and the generalizability of their conclusions to the broader population remains to be validated. By optimizing the study design and expanding the diversity of the sample, this study provides an in-depth analysis of the key inflection points and their dynamic patterns, offering more detailed evidence for elucidating the mechanisms underlying the associations between LE8, LC9, and frailty. Frailty is closely related to depressive symptoms. Research has indicated that the condition of frailty is notably more frequent among older adults with depression (Lohman et al., 2017), with approximately 40.4% of geriatric patients with depression qualifying as pre-frail or frail (Soysal et al., 2017). Frailty is a significant cause of falls in elderly individuals with depression (Lohman et al., 2022).

The association between the LC9 score and frailty remains to be fully understood, though a review of current literature indicates that hormonal fluctuations and inflammation could be pivotal factors. Research indicates that systemic inflammation is a pivotal mechanism contributing to frailty (Marcos-Perez et al., 2020). A balanced diet, which includes anti-inflammatory and antioxidant compounds, optimal fatty acid levels, increased dietary fiber, and a variety of fruits, vegetables, and high-quality proteins, can help mitigate inflammation and oxidative stress (Calder, 2010; Hu and Willett, 2002). These dietary components, rich in antioxidants, protect cells from free radical damage and maintain cellular function, which is essential for preventing frailty associated with cellular decline (Zhang, 2019). Higher levels of physical activity are correlated with lower levels of circulating inflammation (Hjelstuen et al., 2006). Obesity, smoking, inadequate sleep, and excessive sugar intake can elevate the number of free radicals in the body, causing endothelial dysfunction and promoting an inflammatory state (Rajendran et al., 2013). Imbalances in BMI and lipid profiles lead to the infiltration of pro-inflammatory cytokines in adipose tissue, disrupting muscle equilibrium and causing sarcopenia and compromised regenerative capabilities (Batsis and Villareal, 2018). Conditions such as hypertension, diabetes, smoking, and hypercholesterolemia foster a pro-inflammatory state by increasing the expression of inflammatory cytokines (Rajendran et al., 2013). Systemic inflammation is a significant element in the pathogenesis of depression (Ghazizadeh et al., 2020). Factors such as diet, physical activity, smoking, sleep patterns, BMI, lipid profiles, blood pressure, and blood glucose levels can exacerbate inflammatory processes, thereby fueling the development of both depression and frailty. Depressive symptoms, acting as a risk factor for frailty, can further intensify the severity of frailty.

The diminished secretion of testosterone and estrogen in both men and women may impact the loss of muscle mass (Mishra et al., 2019). Hormonal variations associated with aging, including changes in growth hormone, testosterone, thyroid hormone, and insulin-like growth factors, contribute to the loss of muscle mass and strength (Ryall et al., 2008). The influence of education level on the LC9-frailty relationship may be attributed to higher health literacy among individuals with more education. Moreover, they typically have better access to economic and healthcare resources, enabling them to optimize health indicators. This may account for the observed variations in the subgroup analyses.

Our study is underpinned by data from the NHANES database, which is highly regarded for its meticulous data collection methods and large participant volumes, ensuring the robustness and dependability of our findings concerning frailty. By conducting stratified analyses, we have delved into the relationship between LC9 scores and frailty, scrutinizing how this association fluctuates among diverse demographic groups. Nonetheless, our research is not without its intrinsic limitations. Firstly, the cross-sectional nature of our study precludes the confirmation of a causal link between frailty and LC9 scores, emphasizing the requirement for longitudinal research to ascertain causality and the temporal sequence of events. Secondly, despite accounting for a multitude of covariates, there may remain unmeasured confounding factors (such as genetic factors and environmental exposures) that could influence the relationship between LC9 scores and frailty. Thirdly, variations in socioeconomic status and healthcare accessibility could also potentially skew the study’s outcomes. Future research may elucidate the causal mechanisms and temporal dynamics of the relationship between LC9 scores and frailty through longitudinal studies.

## 5 Conclusion

In conclusion, our study reveals a significant curvilinear relationship between the LC9 and LE8 scores and frailty, with LC9 demonstrating a more pronounced and extensive impact. The higher inflection point of LC9 suggests its increased effectiveness in reducing frailty risk over a wider range compared to LE8. Clinically, the LC9 score’s integration of mental health offers a more comprehensive frailty predictor, emphasizing the need for holistic healthcare strategies that address both physical and psychological aspects of health. This study underscores the potential utility of the LC9 score in clinical settings for targeted interventions to prevent or delay frailty, highlighting the importance of considering mental health in frailty management. Further research is necessary to fully realize the practical applications of these findings in improving patient outcomes and quality of life.

## Data Availability

Publicly available datasets were analyzed in this study. This data can be found here: www.cdc.gov/nchs/nhanes/Default.aspx.

## Glossary

NHANES: National Health and Nutrition Examination Survey
LC9: Life’s Crucial 9
LE8: Life’s Essential 8
LS7: Life’s Simple 7
AHA: American Heart Association
BMI: Body Mass Index
PHQ-9: Patient Health Questionnaire-9 (a tool for measuring depression)
HEI-2015: Healthy Eating Index 2015
HbA1c: Hemoglobin A1c
OGTT: Oral Glucose Tolerance Test
CVD: Cardiovascular Disease
CDC: Centers for Disease Control and Prevention
